# EgyPLI: A Real-life Annotated Image Dataset for Egyptian Plant Leaf Identification

**DOI:** 10.1038/s41597-025-06539-8

**Published:** 2026-02-06

**Authors:** Amany M. Sarhan, Mahmoud A. Shaheen

**Affiliations:** 1 Department of Computer and Control Engineering, Faculty of Engineering, Tanta University, Tanta, Egypt; 2 Faculty of Engineering, Elsewedy University for Technology, Cairo, Egypt; 3 Department of Artificial intelligence, Faculty of Artificial intelligence, Delta University for Science and Technology, Gamassa, Egypt

**Keywords:** Agriculture, Electrical and electronic engineering

## Abstract

The Egyptian Plant Leaf Image Dataset (EgyPLI) is the first comprehensive collection of plant leaf images curated in Egypt to support research in automated plant identification. It addresses the lack of locally representative datasets and the broader need for geographically diverse data to enable the development of generalized models. EgyPLI contains real-world leaf images captured under varying viewpoints, lighting conditions, and background clutter, reflecting realistic agricultural environments. Unlike laboratory-controlled datasets, it includes natural noise and variability, supporting the training of robust deep learning models suitable for real deployment. The dataset is carefully annotated and preprocessed to establish a consistent standard for plant identification tasks. EgyPLI comprises 3,588 images covering eight widely cultivated plant species: apple, berry, fig, guava, orange, plum, persimmon, and tomato, including both healthy and diseased leaves. This diversity supports classification, diagnosis, and health assessment applications. To demonstrate its effectiveness, the dataset was evaluated using ResNet50, VGG16, and a custom CNN, achieving accuracies of 61.67%, 96.81%, and 99.22%, respectively. As an available resource, EgyPLI fills a critical gap.

## Introduction

Building a dataset is a fundamental step in advancing research across various domains, as it provides a structured and reliable source of information for training and evaluating machine learning models. A well-organized dataset enhances the accuracy and robustness of AI-driven applications by ensuring models generalizability by learning from diverse, high-quality, and representative data. It enables researchers to develop innovative solutions tailored to specific problems, whether in healthcare, agriculture, environmental monitoring, or any other field. Furthermore, datasets facilitate benchmarking and comparison of different models, allowing researchers to assess performance objectively and refine their approaches. The availability of a dataset also promotes collaboration within the scientific community, accelerating progress by providing a common foundation for multiple studies.

However, the process of collecting image datasets, particularly in agriculture, demands significant effort and manpower, making the process costly. Therefore, sharing these datasets benefits researchers by providing access to diverse data that vary across regions, seasons, soil types, and weather conditions. In many countries, researchers rely on public datasets rather than creating their own. However, given the differences in climate and cultivation practices between regions, they often assume that these datasets are suitable for their local environment, which is not always accurate. Having multiple datasets on the same topic from different countries offers several key benefits. First, it allows researchers to test the robustness of identification models against variations in data. Second, combining datasets from various sources can create a larger, more diverse dataset, enhancing model accuracy and improving generalizability. Additionally, comparing datasets helps scientists determine whether the same object exhibits consistent features across different regions or if variations exist, prompting further investigation into the causes of these differences. Additionally, this dataset contributes to biodiversity conservation by tracking plant populations, identifying endangered species, and studying the impact of environmental changes.

Hence, the development of a specialized dataset directed to Egyptian plant leaves addresses this critical gap and supports a wide range of agricultural, ecological, and botanical research applications. Such dataset enables the development of AI models for accurate plant identification and classification, assisting researchers, botanists, and farmers in distinguishing various species efficiently. It also plays a significant role in precision agriculture by supporting crop health monitoring, detecting diseases at early stages, and optimizing resource management, such as water and fertilizers. Moreover, automated disease recognition through deep learning minimizes reliance on expert intervention, making plant health management more accessible and cost-effective. It also facilitates plant phenotyping by allowing AI systems to estimate leaf counts, monitor growth stages, and predict crop yields, helping farmers make data-driven decisions. Unlike global plant datasets, which often lack region-specific representation, a specialized dataset tailored to Egyptian flora bridges this gap, ensuring that AI models are adapted to local agricultural conditions.

There are numerous datasets for plant leaves identification, for example: Plant Village^1^ introduces an open-access repository of over 50,000 curated images of healthy and diseased crop leaves, hosted on the PlantVillage platform. This dataset, labeled by plant pathology experts, aims to facilitate the development of mobile disease diagnostics using machine learning and crowdsourcing. The data is openly licensed under Creative Commons to ensure accessibility and promote collaborative innovation in agricultural diagnostics. Plant Leaves^2^ introduces a database of 4503 leaf images from twelve plant species (Mango, Arjun, Alstonia Scholaris, Guava, Bael, Jamun, Jatropha, Pongamia Pinnata, Basil, Pomegranate, Lemon, and Chinar) in both healthy and diseased conditions. Collected at Shri Mata Vaishno Devi University from March to May 2019, the images were captured using a Nikon D5300 camera in a closed, Wi-Fi-enabled environment. PlantDoc^3^ is a dataset aims to facilitate the development of accurate and efficient plant disease detection methods using computer vision techniques. The resource is intended to help researchers and practitioners create solutions that can automatically identify and classify plant diseases from images, contributing to improved crop management and agricultural productivity.

In^4^, a deep learning framework is introduced for tomato leaf disease detection on Tomato Leaves dataset. The authors developed a convolutional neural network (CNN)-based model trained on labeled images of tomato leaves, focusing on distinguishing between healthy and diseased specimens. Their approach likely leveraged standard datasets like PlantVillage and incorporated techniques such as image preprocessing or data augmentation to handle variability in leaf appearances. Other datasets, such as Citrus Leaves^5^, Leaf1^6^, Flavia^7,8^, Middle European Woody Plants^9^, MalayaKew (MK) Leaf^10^, Amazon Forest^11^, Swedish Leaf^12^, and LeafSnap^13^, have also contributed to plant classification and species identification using machine learning and deep learning approaches.

Despite Egypt being one of the leading agricultural countries in the whole world since the ancient days, there is currently no dedicated dataset for Egyptian plant leaves. According to the Food and Agriculture Organization (FAO), Egypt cultivates over 120 important crop types, that contributes to approximately 11.3% of the country’s GDP, in addition to incorporate around 24% of the Egyptian labor force. Given Egypt’s diverse plant species and unique environmental conditions, the absence of such a dataset limits research advancements in plant identification, disease detection, and precision agriculture. Developing a comprehensive dataset of Egyptian plant leaves would not only support deep learning applications in agriculture but also enable researchers to analyze plant health, improve crop management strategies, and contribute to biodiversity conservation. By filling this gap, researchers can ensure that AI models are better adapted to Egypt’s specific agricultural landscape, rather than relying on datasets from other regions that may not accurately reflect local plant species and conditions. Moreover, this dataset can be utilized in many scientific directions as explained above.

Compared to other plant leave dataset, such as PlantVillage, PlantDoc, and LeafSnap, EgyPLI features leaves collected in natural outdoor settings with varying illumination, background clutter, and occlusions—conditions not fully reflected in PlantVillage or LeafSnap. EgyPLI contains species unique to Egypt that are not found in PlantVillage or PlantDoc, making it useful for region-specific plant disease and categorization studies. EgyPLI images capture leaves in natural orientations, mixed foliage, and real-farm settings, overcoming the constraints of synthetic or background-removed datasets such as PlantVillage. While PlantVillage focuses on disease classification in controlled environments, EgyPLI is intended to enable detection and segmentation activities in the field, supplementing rather than replacing current datasets.

The main goals of this paper are:

1. Introducing a novel in-field image dataset for Egyptian plant leaves - Egyptian Plant Leaf Image Dataset (EgyPLI)^14^ - that contains 3,588 images representing 8 plant species. The data is collected under a natural, uncontrolled environment (i.e., in-field conditions) to mimic real-world situations, guaranteeing its significance for real-world agricultural applications.
2. Performing a comprehensive analysis for the dataset to ensure its quality, completeness and annotation correctness for deep learning possible application.
3. Training and assessing various deep learning models (including custom and pretrained models) using the collected dataset to evaluate their performance and reporting their results.

The rest of the paper is organized as follows: section 2 shows the methodology followed to collect and organize the dataset, section 3 shows the results of training and validating the deep learning model on the dataset. Section 4 gives the conclusion and future work.

## Methodology

To capture real-world complexities, the dataset includes high-resolution images that provide clear and detailed visuals of in-field Egyptian plant leaves. Covering a wide range of backgrounds, lighting conditions, and angles, the dataset contains images of plant leaves. This diversity enhances the robustness of deep learning models for real-world applications.

**Fig. 1:**
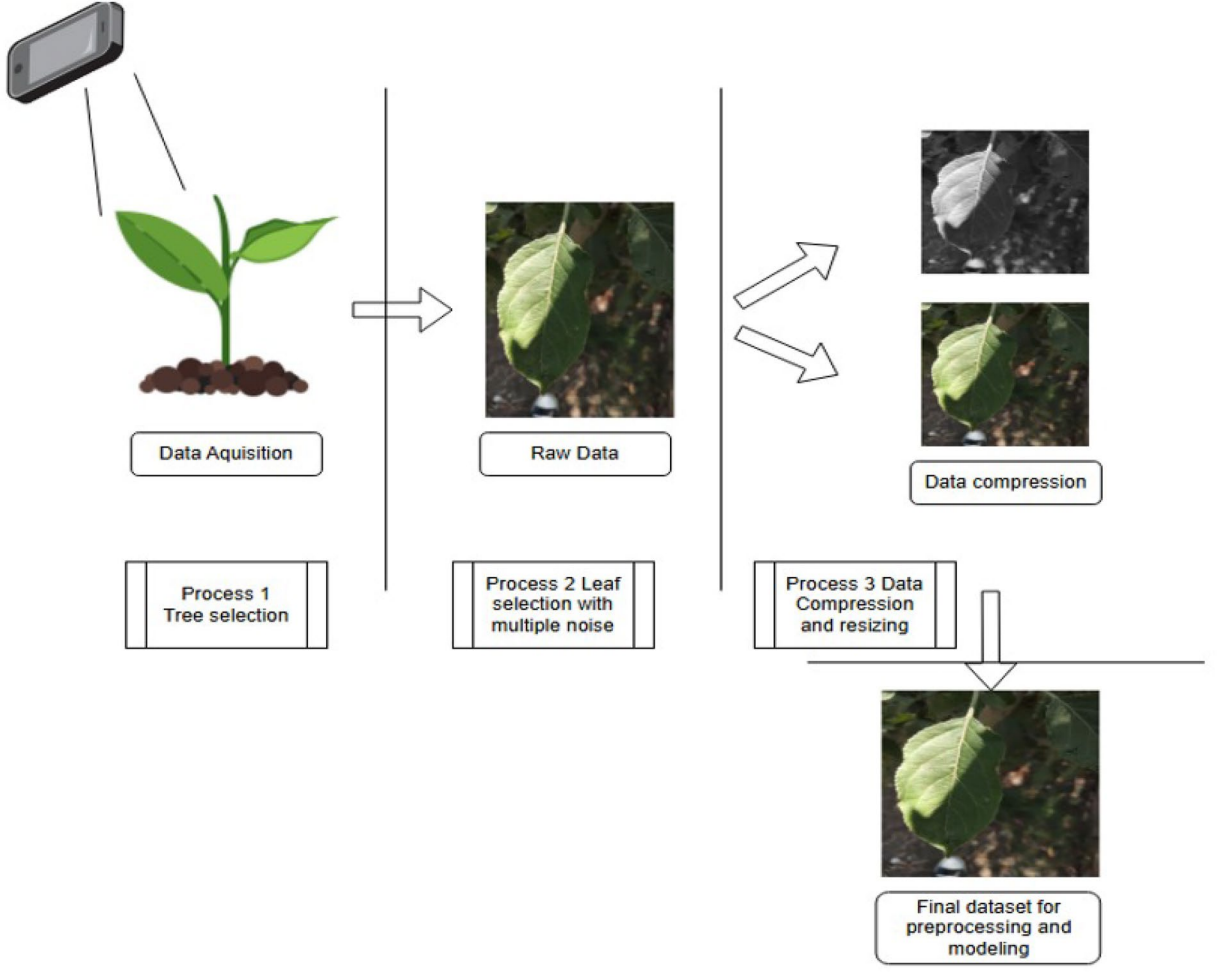
Dataset collection pipeline.

Figure 1 represents a structured process for acquiring, processing, and preparing plant leaf images for further analysis and modeling. The process begins with data acquisition, where a camera or smartphone captures images of a selected tree leaves. This is labeled as “Process 1: Tree Selection and Image Capturing”, ensuring that the images are taken from the appropriate plants for the intended study, such as disease detection or classification tasks. Once the images are captured, they are stored as raw data, which contains various elements like leaves, background noise, lighting variations, and other environmental factors.

In process 2: “Leaf Selection with Multiple Noise”, specific leaf images are chosen from the raw data, acknowledging the presence of natural noise like shadows, uneven lighting, or background elements. This step is crucial in ensuring that the dataset represents real-world conditions and is not artificially controlled, as in lab-based datasets. The selected images then undergo process 3: Data Compression and Resizing, which involves reducing the image size while maintaining quality. This resizing step was performed to ensure compatibility with widely used CNN architectures, which typically require fixed-size inputs (e.g., 224 × 224 or 256 × 256 pixels). This operation reduces memory usage and training time without altering the visual characteristics required for classification. The process may include grayscale conversion or maintaining color images, depending on the application’s needs.

The final outcome is a dataset ready for preprocessing and modeling, which can be further refined through techniques such as image augmentation, segmentation, or feature extraction. This structured pipeline is essential for building robust models capable of real-world performance. All images are preserved in full-color RGB to retain texture and color information, which are essential features for plant leaf identification.

### Data acquisition

The dataset was collected over a two-month period, from July 2024 to August 2024, in Abu-Mustafa Village, Kafrelsheikh, Egypt. The region is known for its agricultural diversity, making it an ideal location for real-world plant data collection. The images were captured using a Xiaomi M2012K11AG (Poco F3) smartphone, which features a 5 mm focal length lens, a maximum aperture of f/1.8, and ISO 76 settings, ensuring high-quality image acquisition under natural lighting conditions. The dataset consists of 3,588 images originally captured at a high resolution of 3000 × 4000 pixels in JPG format, covering eight plant species: Apple, Berry, Fig, Guava, Orange, Plum, Persimmon, and Tomato. To ensure compatibility with deep learning models and reduce computational load, all images were later resized to a standardized resolution of 256 × 256 pixels before being made publicly available.

**Fig. 2:**
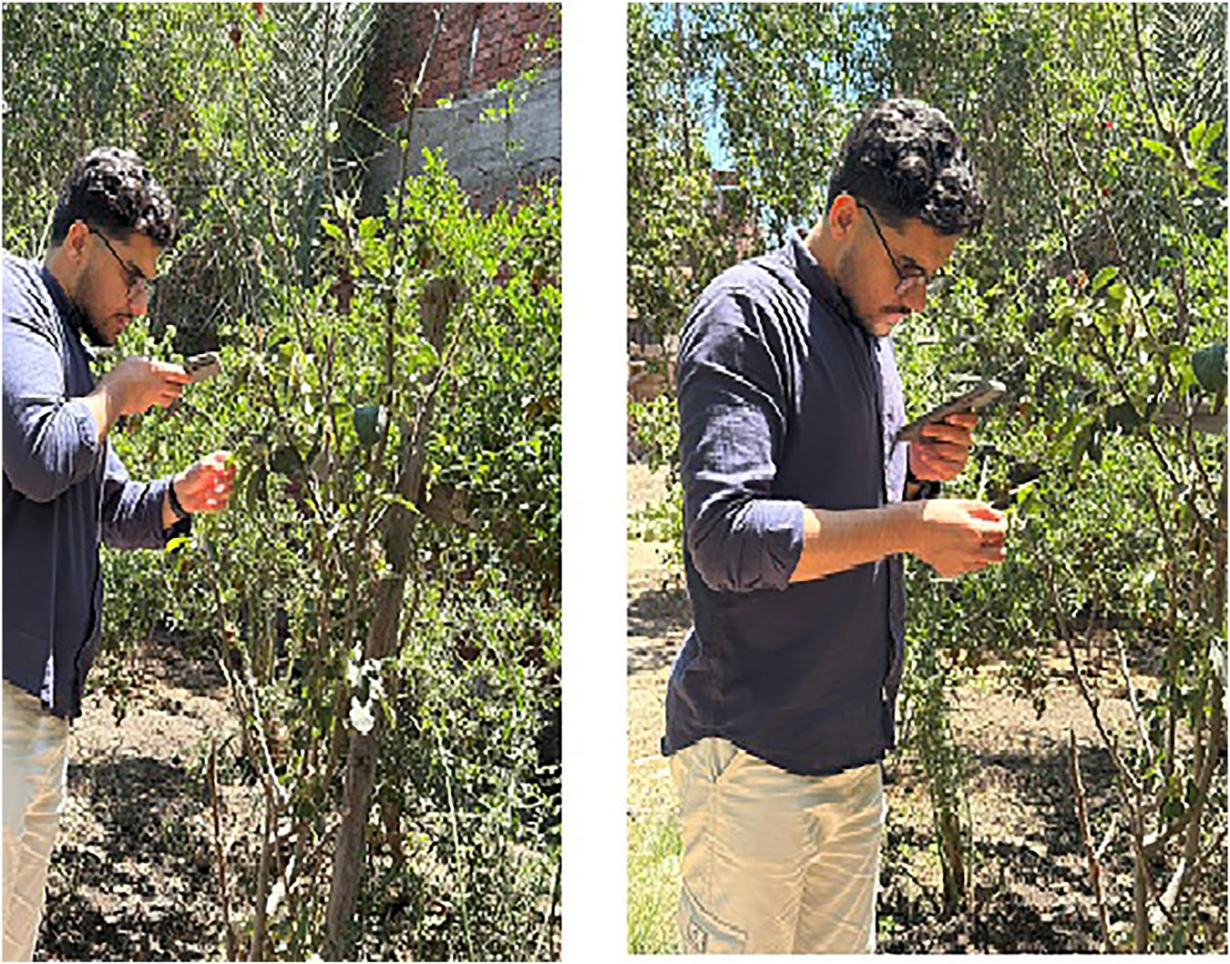
Capturing data process for the Egyptian Plant Leaf Image Dataset in-filed real-life images*. *The individual shown in this figure is one of the authors and has provided informed consent for the publication of this image.

**Fig. 3:**
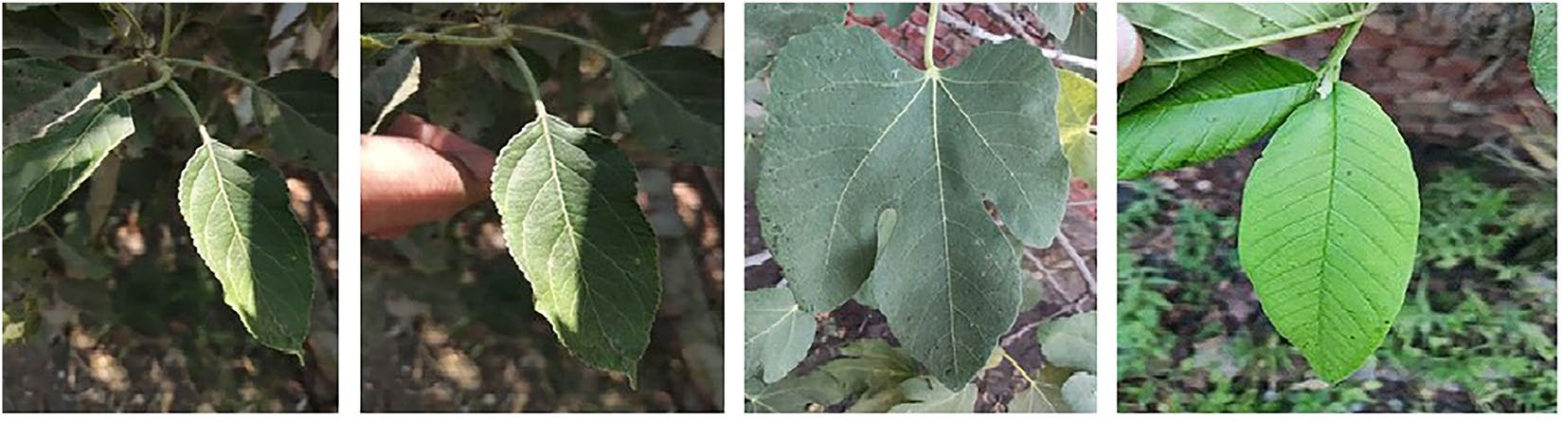
Samples from various classes in the Egyptian Plant Leaf Image Dataset showing different times of day and plant appearance.

Our dataset images for plant leaves are collected under natural, uncontrolled conditions, making it more diverse, comprehensive, and representative of real-world scenarios (as shown in Fig. 2). Unlike datasets captured in controlled laboratory settings, this dataset represents real-world agricultural conditions, including natural occlusions, variations in lighting, and environmental noise. The images were taken at different times of the day to capture diverse illumination conditions and plant appearances as shown in Fig. 3. Manual data acquisition was performed to ensure that each species was well-represented across different growth stages and environmental backgrounds. This enhances the dataset’s robustness for machine learning applications, including plant classification, disease detection, and crop monitoring.

To ensure high-quality data, pre-processing steps such as data compression and resizing were applied while preserving image clarity. The images were then classified into eight distinct classes, corresponding to the respective plant species. Multiple deep learning models will be applied to evaluate the dataset’s effectiveness in AI-driven agricultural solutions. The dataset serves as a valuable resource for researchers developing AI-based plant identification and disease detection systems, bridging the gap between controlled experimental datasets and real-world agricultural challenges.

The EgyPLI dataset^14^ is gathered in-field under natural, uncontrolled conditions, making it more diverse, comprehensive, and representative of real-world scenarios. It captures the true complexity of plant structures, providing realistic visual information similar to what an operator would observe in the field. In contrast, models trained solely on controlled-condition images may struggle to generalize when exposed to complex environments with intricate leaf structures. We intentionally incorporated diverse backgrounds, lighting conditions (e.g., direct sunlight, shade), and viewing angles in order to simulate real-life complexities relevant for deep learning-based classification and detection tasks. In such manner, this dataset is ideal for developing autonomous intelligent systems that require adaptability and robustness in real-world agricultural applications.

**Fig. 4:**
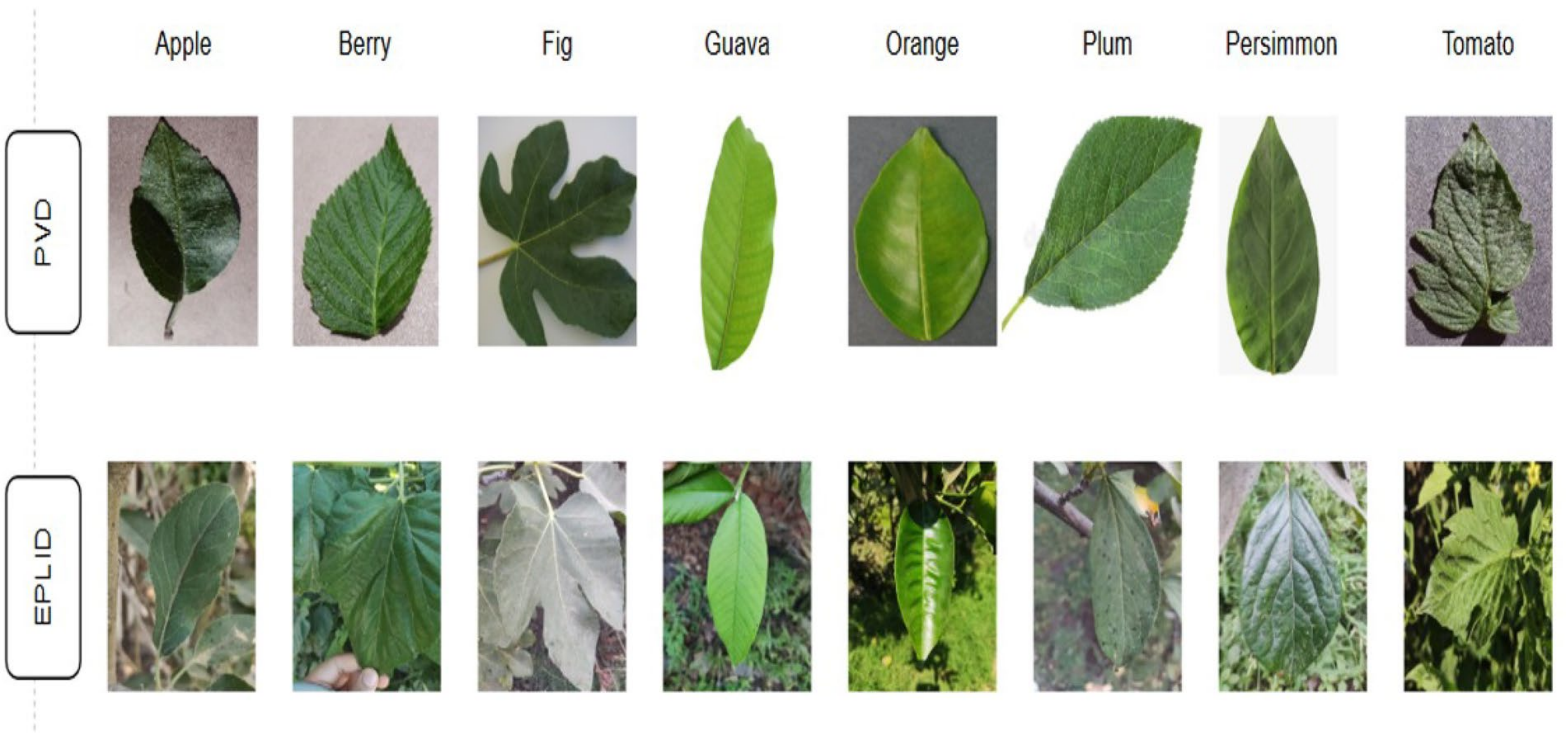
Samples from various classes in the Egyptian Plant Leaf Image Dataset show the gap between lab-controlled and real-life images.

Compared to the Plant Village dataset that contains images taken under controlled settings, our dataset is more reliable and realistic. The Plant Village dataset limits the efficiency of detecting plant classes because, in reality, plant images may contain multiple leaves with different types of background conditions at varying lighting conditions. Samples of both datasets are shown in Fig. 4, which show the difference between the data collection condition.

### Dataset description

The dataset originally consisted of 3,588 high-resolution images captured at 3000 × 4000 pixels in JPG format. For the publicly released version and model training purposes, all images were resized to a standardized resolution of 256 × 256 pixels while preserving the RGB color information. The dataset covers eight plant species: Apple, Berry, Fig, Guava, Orange, Plum, Persimmon, and Tomato

These images were collected using a Xiaomi Poco F3 smartphone from Kafrelsheikh, Egypt, an agriculturally rich region known for diverse crop production. Unlike existing datasets that primarily rely on laboratory-controlled images, this dataset represents real-world conditions, capturing variations in lighting, leaf occlusions, background noise, and natural environmental factors. Such diversity enhances the dataset’s robustness for training AI models capable of handling field-level complexities.

Each image is categorized into one of eight classes, making it suitable for classification tasks, plant recognition, and disease detection. The high-resolution format ensures that fine details, such as leaf texture and vein patterns, are preserved, which is essential for deep learning models. Furthermore, multiple machine learning models will be applied to assess its performance across different architectures, enabling comparisons between CNN-based and transformer-based vision models. Table 1 provides images distribution across plant categories along with their scientific names. This dataset contributes to the field of precision agriculture and automated plant monitoring, supporting the development of AI-driven applications for farmers, researchers, and agronomists. The availability of high-quality real-world images fills the gap between controlled laboratory datasets and practical agricultural implementations, making it a valuable resource for advancing computer vision applications in plant science.

**Table 1:** Plant images distribution across plant categories.

Class Name	Scientific name	No. of images	Representation
**Apple**	Malus domestica	519	14.46%
**Berry**	Rubus idaeus	340	9.47%
**Fig**	Ficus carica	508	14.15%
**Guava**	Psidium guajava	520	14.49%
**Orange**	Citrus sinensis	547	15.24%
**Plum**	Prunus domestica	468	13.04%
**Persimmon**	Diospyros kaki	527	14.68%
**Tomato**	Solanum lycopersicum	159	4.43%
**Total**		3,588	100%

## Data Records

**Fig. 5:**
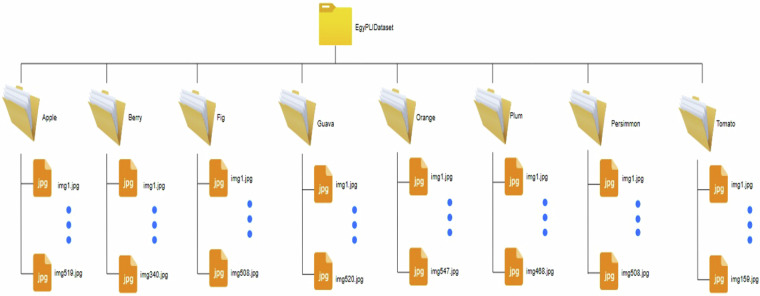
Folder structure of the EgyPLI dataset^14^.

The dataset is available at Kaggle repository^14^ with the name EgyPLI dataset. The dataset is distributed under the CC BY 4.0 license, allowing unrestricted reuse provided proper attribution is given. The dataset is organized into a main directory titled “EgyPLIDataset”, which contains eight subfolders, each representing one plant species. Each folder contains the corresponding leaf images (Apple, Berry, Fig, Guava, Orange, Plum, Persimmon and Tomato) named using a standardized naming system to ensure consistency and easy indexing (as illustrated in Fig. 5).

### Data overview

The dataset consists of 3,588 real-world leaf images captured under natural environmental and lighting conditions. All images are stored in JPG format and standardized to a resolution of 256 × 256 pixels for compatibility with deep learning applications. To ensure accurate species labelling, a structured annotation workflow was followed. Each image was initially labeled by trained annotators and subsequently reviewed and validated by an agricultural expert specializing in crop morphology. Species identification was performed based on morphological characteristics including leaf shape, venation pattern, margin type, colour, and surface texture. This two-stage verification process ensured high labelling reliability. The provided annotations indicate the plant species and whether the leaf is healthy or diseased. The dataset includes classification labels only and does not currently provide bounding boxes or segmentation masks. However, the natural scene images make the dataset suitable for future segmentation and object detection extensions.

The collected dataset, along with the annotation analyzed in this study, is currently available online for public usage. It can be downloaded from Egyptian Plant Leaf Image dataset (EgyPLI) at^14^. The Egyptian Plant Leaf Image dataset is a collection of high-quality images focusing on various plant leaves, aimed at supporting research and development in plant health monitoring, disease detection, and species identification. This dataset contains images that capture different plant species under varying conditions, allowing for diverse applications in agriculture, botany, and AI-based plant recognition.

The dataset consists of 3,588 high-resolution images from 8 Egyptian plant crops. Image files are in standard formats (e.g., JPG or PNG). Organized into folders based on plant type or condition for easy access and utilization. This dataset is ready for integration into machine learning pipelines for training and evaluation in various agriculture and plant-related AI applications, allowing for easy interaction with deep learning frameworks like PyTorch and TensorFlow.

## Technical Validation

To validate the quality and usefulness of the dataset, several advanced deep learning models are implemented for classification. The performance of these models is evaluated using accuracy, precision, recall, and F1-score metrics. The processed dataset was used to train several deep learning models, including: Custom CNN, ResNet50 and VGG16. Each model was trained on the training dataset and tested on validation and test datasets to measure how well it could identify and classify plant leaves. The primary goal to train such models is to establish trustworthy baseline performance that could guide future researchers working with this dataset and to ensure that this dataset is good enough to be used by deep learning models.

By using validation properly, researchers can develop a model that is more accurate, reliable, and useful in real-world applications. Other deep learning architectures, such as EfficientNet and Vision Transformers, could also be applied for classification tasks. However, in this study, we focus on three models in our work — Custom CNN, ResNet50, and VGG16 — planning to explore additional models in future work.

### Models details

**Fig. 6:**
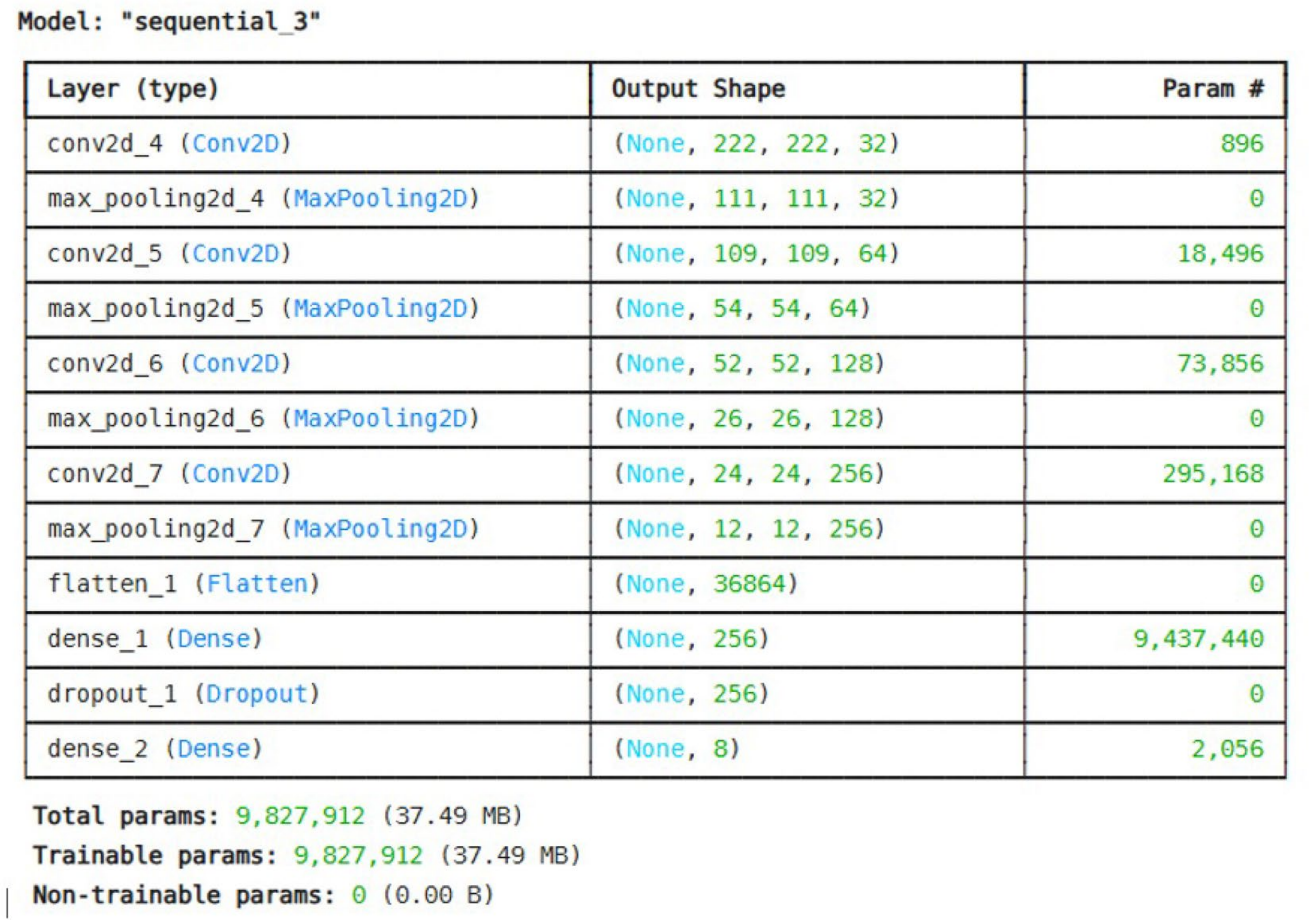
Custom CNN architecture.

To assess the dataset’s ability to enhance machine learning model performance in plant identification (classification), we conducted experiments using a custom CNN, and established CNN-based pre-trained models namely; ResNet50^15^ and VGG16^16^ for the classification analysis of the eight classes, CNN hits the highest score as shown in Table 2. The custom CNN architecture is shown in Fig. 6.

**Table 2:** Model specifications.

Model	Epochs	Validation Loss	Accuracy	F1-score	Recall
**ResNet50**	50	1.3538	61.67	58.00	60.00
**VGG16**	50	0.2413	96.81	96.00	96.00
**Custom CNN**	50	0.0308	**99.22**	**99.00**	**98.00**

### Evaluation metrics

To evaluate the performance of the models, the following metrics are used: accuracy, precision, recall, and F1-score which are computed as follows:

Accuracy (the ratio of true positive predictions to the total predicted positives) is crucial in this context as it indicates how many of the predicted plant species were actually positive^17^.

1 $${\rm{Accuracy}}=\frac{{\rm{True\; Positives}}}{{\rm{True\; Positives}}+{\rm{False\; Positives}}}$$

Recall (the ratio of true positives to the total actual positives) is important to ensure that the model detects the maximum number of plant types, especially in critical agriculture applications^17^.

2 $${\rm{Recall}}=\frac{{\rm{True\; Positives}}}{{\rm{True\; Positives}}+{\rm{False\; Negatives}}}$$

The F1-score integrates both precision and recall measures, offering a balanced evaluation of both metrics. It is especially beneficial for handling imbalanced datasets, such as those used in plant identification^17^.

3 $${\rm{F}}1 \mbox{-} {\rm{score}}=2\times \frac{{\rm{Precision}}\times {\rm{Recall}}}{{\rm{Precision}}+{\rm{Recall}}}$$

### Performance results and analysis

The model performance of the EgyPLI dataset^14^ is reported in Table 3. These models have been developed and trained on a device with 16 GB RAM, Ryzen7, and GPU Nvidia GEFORCE RTX 3050 with 4 GB RAM connected to Kaggle environment on TPU T100. We evaluated the three deep learning models by using 3,588 plant leaf images, 2,870 images for training, and the test set contains 718 images.

**Table 3:** Class-wise Precision, Recall, and F1-score for the three evaluated deep learning models.

	Precision			Recall			F-1 score		
	ResNet50	VGG16	CNN	ResNet50	VGG16	CNN	ResNet50	VGG16	CNN
Orange	0.74	0.98	**0.98**	0.73	0.96	**1.00**	0.74	0.98	**0.99**
Berry	**1.00**	0.93	0.99	0.01	0.97	**0.99**	0.03	0.95	**0.99**
Tomato	0.68	0.88	**1.00**	0.70	**0.97**	0.93	0.69	0.92	**0.97**
Fig	0.68	0.98	**0.99**	0.75	0.98	**0.98**	0.71	0.98	**0.99**
Apple	0.52	0.92	**0.99**	0.73	0.96	**0.99**	0.61	0.94	**0.99**
Guava	0.43	0.99	**1.00**	0.52	0.97	**0.99**	0.47	0.98	**0.99**
Persimmon	0.68	0.98	**1.00**	0.57	0.95	**1.00**	0.62	0.97	**1.00**
Plum	0.59	0.94	**0.98**	0.66	0.92	**0.99**	0.62	0.93	**0.98**

We trained both networks by using the same parameters. These training parameters are, epoch size is set as 50 in all models, categorical cross entropy selected as loss function, ADAM is used as the optimizer, learning rate set as 0.0005, weight decay set as 0.9 and batch size set as 16. Different approaches were used for avoiding overfitting. Firstly, batch normalization was used after every convolutional layer. Secondly, the dropout method was used after fully connected layers with 0.3 rate. To avoid the risk of overfitting, given that our dataset size is moderate, we applied several data augmentation techniques such as random flipping, rotation, and brightness adjustments during training.

**Fig. 7:**
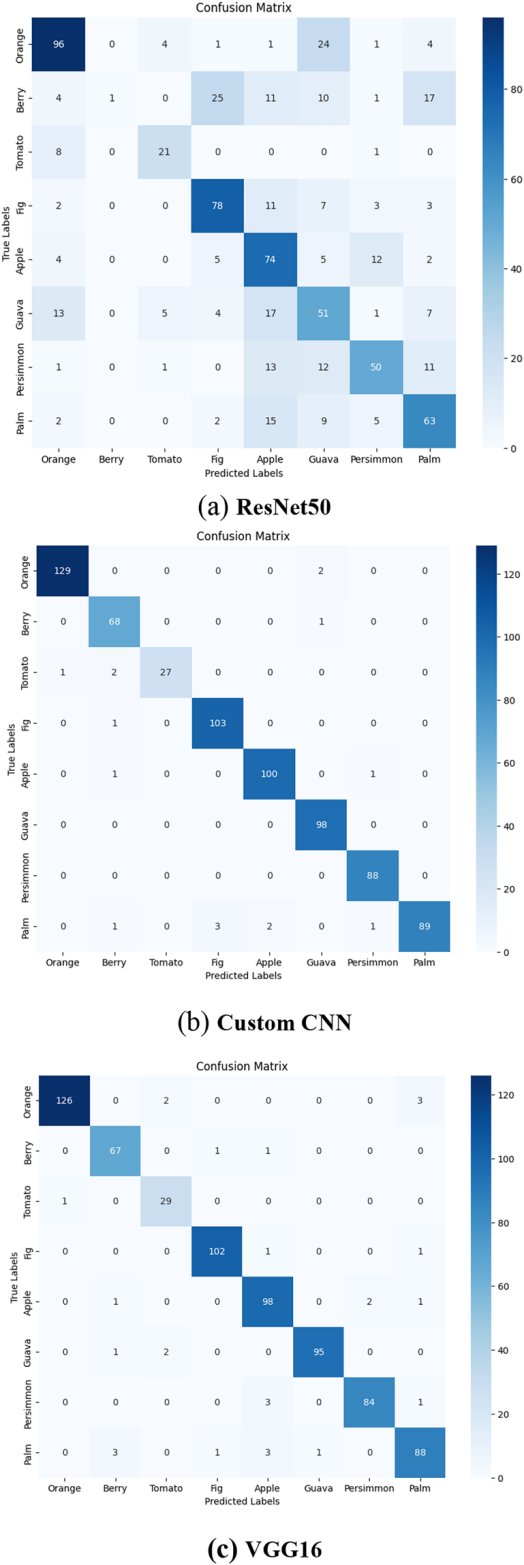
Confusion matrix for the three trained models.

**Fig. 8:**
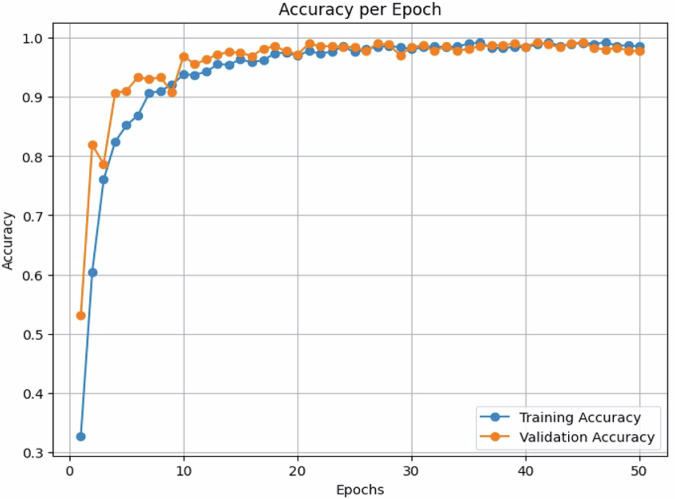
CNN accuracy per epoch.

**Fig. 9:**
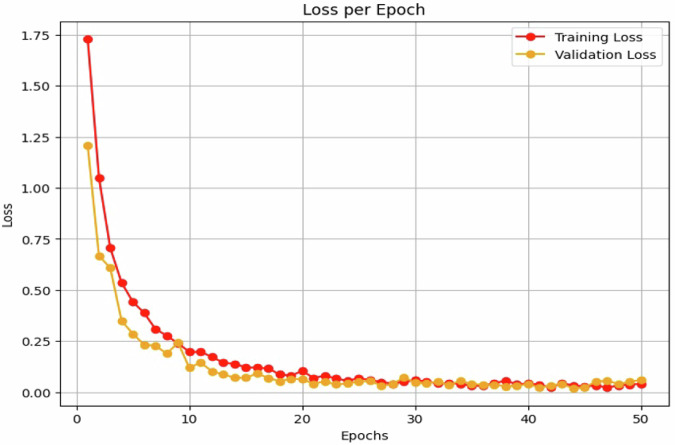
CNN loss per epoch.

The experimental results show that the custom ResNet50 model achieved an overall accuracy of 61.67% on the validation dataset, while VGG16 reached only 96.81% accuracy, and CNN was the highest among the three models with 99.22% accuracy. The confusion matrix is also used to evaluate the model’s ability to classify data correctly, as shown in Fig. 7. Figures 8, 9 show the accuracy per epoch and loss per epoch for CNN model.

Concentrating on the CNN confusion matrix, which yielded the best classification accuracy result, we can see that the diagonal values are consistently the highest in each row, indicating strong true positive rates across most classes. The highest correct classification scores are seen in: Orange (131 correct predictions), Fig (102), Apple (101) and Plum (95).

The number of misclassified leaves are small (e.g., Orange is misclassified as Guava and Plum is misclassified as Fig) as those leaves are visually similar. This reflects the model’s ability to discriminate between classes effectively and the clearance of the dataset images. Two types were the worst results in classification: Tomato and Persimmon, this is due that they have low number of image samples. This will require data balance algorithms via augmentation or through other approaches.

The results in Table 3 show that the VGG16 and CNN models consistently outperform ResNet50 across most evaluation metrics. VGG16 demonstrates high and stable precision and recall values across nearly all plant species, indicating strong generalization and robustness when classifying real-world leaf images. The custom CNN also achieves competitive performance, especially for classes with a balanced number of samples, benefiting from its lighter architecture and adaptation to the dataset characteristics.

In contrast, ResNet50 shows noticeably lower performance across several classes. This reduction in accuracy can be attributed to the model’s deeper and more complex architecture, which requires a larger and more diverse training dataset to avoid underfitting. Since EgyPLI contains a limited number of samples in some classes, such as Tomato and Persimmon, ResNet50 does not fully optimize its high-capacity feature extraction layers. On the other hand, VGG16’s simpler structure and CNN’s compact design enable them to learn more discriminative features effectively from the available data without overfitting.

Furthermore, some classes exhibit high similarity in leaf shape and texture (e.g., Plum and Fig), leading to misclassifications in deeper architectures that rely heavily on fine-grained texture features. The inclusion of class-wise results and confusion matrix confirms that performance challenges are primarily linked to class imbalance and visual similarity, rather than model training configurations.

### Conclusion and Future work

In this paper, we have introduced a new dataset called Egyptian Plant Leaves dataset (EgyPLI) that compromise eight species with their healthy status. We have discussed the dataset details and validated it using three deep learning models: Custom CNN, ResNet50 and VGG16. One of the limitations of our dataset is the class imbalances, particularly in the Tomato class. In this work, we used data augmentation to address this issue. However, other methods, such as class-weighted loss functions, oversampling, or synthetic data creation (for example, SMOTE or GAN-based approaches), could improve model robustness in a future work. Benchmarking against combined datasets (e.g., training on PlantVillage + EgyPLI vs. EgyPLI alone) van be helpful to investigate the potentiality of the introduced dataset.

### Consent Statement

This statement ensures that Eng. Mahmoud Shaheen is approving his images to be published in this paper.

## Data Availability

The data underlying this article is shared at 10.34740/kaggle/dsv/9782525.

## Code availability

The code underlying this article is shared at https://github.com/MahmoudShahenn/EgyPLI.
